# A rare human CEP290 variant disrupts the molecular integrity of the primary cilium and impairs Sonic Hedgehog machinery

**DOI:** 10.1038/s41598-018-35614-x

**Published:** 2018-11-26

**Authors:** Michaela B. C. Kilander, Chun-Hung Wang, Chia-Hsiang Chang, Jonathan E. Nestor, Kevin Herold, Jin-Wu Tsai, Michael W. Nestor, Yu-Chih Lin

**Affiliations:** 1 Program in Neuroscience, Hussman Institute for Autism, Baltimore, MD 21201 USA; 2 Institute of Brain Science, School of Medicine, National Yang-Ming University, Taipei, 112 Taiwan; 3 Taiwan International Graduate Program (TIGP) in Molecular Medicine, National Yang-Ming University and Academia Sinica, Taipei, Taiwan; 4 Brain Research Center (BRC), and Biophotonics and Molecular Imaging Research Center (BMIRC), National Yang-Ming University, Taipei, 112 Taiwan; 5 Program in Molecular Medicine, University of Maryland School of Medicine, Baltimore, MD 21201 USA

## Abstract

The primary cilium is a microtubule-enriched cell-communication organelle that participates in mechanisms controlling tissue development and maintenance, including cerebellar architecture. Centrosomal protein of 290 kDa (CEP290) is a protein important for centrosomal function and ciliogenesis. Mutations in CEP290 have been linked to a group of multi-organ disorders - termed ciliopathies. The neurophysiological deficits observed in ciliopathies are sometimes associated with the progression of autistic traits. Here, the cellular function of two rare variants of CEP290 identified from recent exome sequencing of autistic individuals are investigated. Cells expressing Cep290 carrying the missense mutation R1747Q in mouse exhibited a defective Sonic hedgehog (Shh) signalling response, mislocalisation of the Shh receptor Smoothened (Smo), and dysregulation of ciliary protein mobility, which ultimately disrupted the proliferation of cerebellar granule progenitors (CGPs). This data was furthermore corroborated in an autism patient-derived iPSC line harbouring the R1746Q rare CEP290 variant. Evidence from this study suggests that the R1746Q mutation interferes with the function of CEP290 to maintain the ciliary diffusion barrier and disrupts the integrity of the molecular composition in the primary cilium, which may contribute to alterations in neuroarchitecture.

## Introduction

The primary cilium is anchored to the mother centriole and protrudes from the cell soma of almost every cell in the body^1–3^. The main function of the primary cilium is the regulation of cell division, proliferation, polarity, and migration^4^. The appearance of primary cilium is dynamic and intimately associated with cell division and cell cycle progression^5^. Numerous studies have shown that mutations in ciliary proteins often affect the process of ciliogenesis and/or the structural integrity of the cilium, resulting in devastating consequences to the cell^6^. In a neurobiological context, ciliary signalling plays an important role in the events leading up to the establishment of proper neuroarchitecture. This includes the proliferation, differentiation, migration, and neurite outgrowth of neurone progenitors and mature neurones^7–13^. Recent studies found that primary cilium-coordinated signalling plays a role in the development of cortical and striatal neuronal circuits by regulating dendrite arborisation and synaptic stability in parvalbumin and somatostatin-positive GABAergic interneurones, suggesting a contribution of primary cilia in the functional specification of neurones^14,15^.

Centrosomal protein of 290 kDa (CEP290) is a protein that plays an important role in the formation and stabilisation of the primary cilium as well as centrosomal function^16,17^. This has been demonstrated by a consistent reduction in the number of ciliated cells in RNAi-mediated CEP290 knockdown cultures^18–20^. CEP290 has also been shown to control the molecular integrity of the primary cilium by acting as a central component of the ciliary diffusion barrier located at the transitional zone^16,21–25^. Numerous mutations of CEP290 have been identified as associated with a group of multi-organ disorders called ciliopathies^19,26–29^. Although the neurophysiological deficits observed in ciliopathies are not well-understood, some are associated with the progression of Autism Spectrum Disorder (ASD)^30–32^. ASD comprises a range of neurological conditions characterised by qualitative differences in communication and social interaction^33,34^. Despite several decades of research, the underlying cause of autism remains elusive due to the heterogeneity of individuals and their genetic backgrounds^35,36^. Recent exome sequencing of a cohort of autistic individuals found two rare variants in CEP290^37^. Here, the impacts of these two CEP290 variants on cilia-related cellular processes and signalling were investigated. Assessments of cilia morphology, ciliary Shh signalling and ciliary protein dynamics were performed using NIH-3T3 cells, a well-established model system for the study of primary cilia. In addition, human induced pluripotent stem cells (hiPSCs) derived from individuals with autism containing a CEP290 variant were used. In order to gain further understanding of the potential effect during neurodevelopment *in vivo*, CEP290 function in the proliferation of cerebellar granule progenitors was examined.

## Results and Discussion

### CEP290 variants are expressed normally and do not affect primary cilium stability and morphology in quiescent NIH 3T3 cells

**Figure 1:**
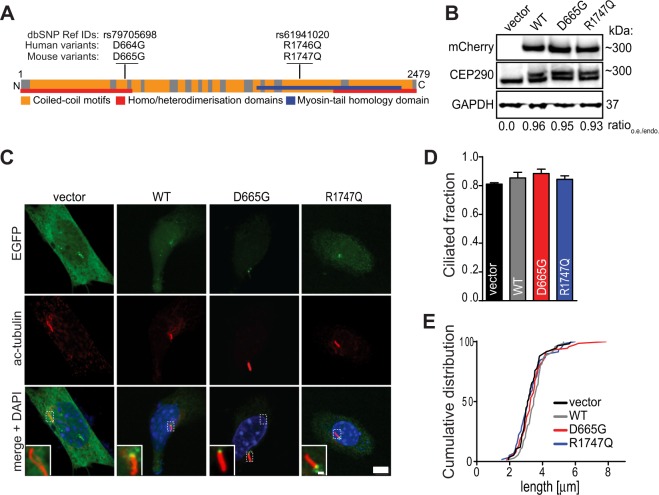
Cep290 mutant variants are expressed normally and do not affect cilia morphology in quiescent cells. (**A**) A schematic representation of the full length CEP290 protein shows positions of autism-associated variants in human and corresponding mutations in mouse. (**B**) Whole cell lysates of NIH/3T3 cells transfected with indicated constructs were analysed by Western blot probed with a CEP290 antibody recognising both endogenous and overexpressed Cep290 proteins; note appearance of dual bands only in samples transfected with mCherry-Cep290 plasmids. Membrane was stripped and re-probed with antibody targeting mCherry. Images show cropped blots; see also Fig. S4A–C. Levels of protein expression were determined by measuring optical density of bands detected by CEP290 antibody and reported as the ratio of overexpression (o.e.; upper band) to endogenous expression (endo.; lower band). Values show the means from four independent experiments, GAPDH was used as loading control. (**C**) NIH/3T3 cells were transfected with indicated EGFP-Cep290 constructs and starved for 24 hrs prior to fixation and immunostaining procedures. Acetylated tubulin (Ac-tubulin) was used to mark the primary cilium. Zoomed-in areas in micrographs indicate location of EGFP-Cep290 WT and mutant proteins at the proximal end of the axoneme. Scale bars = 5 μm and 2 μm. (**D**) Fractions of cilia bearing cells in the transfected cell populations were determined from three experiments, n ≥ 120 cells for each group. (**E**) Cumulative frequency distributions of cilium lengths. Analysis was performed by acquiring 6–10 μm Z-stacks of EGFP positive cells and axoneme lengths were thereafter measured (n ≥ 60 cells/group, three experiments).

Two variants of CEP290 with missense mutations were found in families of autistic individuals not diagnosed with clinical symptoms of ciliopathy (rs79705698, p.D664G and rs61941020, p.R1746Q) (Fig. 1A)^37^. Although the R1746Q variant was previously identified in Joubert Syndrome (JS), an autosomal recessive disorder that is characterised by neurocircuit deficits in the cerebellum^38^, the contribution of this mutation to the disease was predicted to be benign and no further examination was conducted. However, the following experiments will provide evidence suggesting that the R1746Q mutation of CEP290 may generate a deleterious phenotype in a physiological context.

Figure 1A shows a schematic representation of the CEP290 full-length protein and the positions of the mutations within a domain predicted to be involved in homo/heteromerisation (D664G) and in the myosin-tail homology domain (R1746Q). To investigate the cellular and molecular functions that may be altered by these mutations, CEP290 mutants were engineered from a mouse wildtype Cep290 N-terminally EGFP- or mCherry-tagged construct. The sequence of Cep290 has one amino acid shifted downstream in the mouse genome. Therefore, the two corresponding missense mouse mutants of Cep290 are D665G and R1747Q, respectively. To date, the majority of identified CEP290 mutations generate truncated products^29^. Thus, the sizes of the missense mutated proteins were first analysed. When expressing mCherry-tagged constructs in NIH/3T3 cells, no differences in protein expression and migration in SDS-PAGE were observed when comparing the mutants to the wildtype (WT) protein. Moreover, levels of overexpressed proteins were similar across all constructs and were not significantly more expressed than endogenous Cep290 protein (Fig. 1B; Ratio_o.e./endo._).

To first determine whether the Cep290 missense mutations impact cilia formation and/or cilia length, NIH/3T3 cells were serum-starved for 24 hrs to promote the G0 state and induce ciliogenesis. First, the subcellular localisation of transfected EGFP-Cep290 constructs was evaluated. No difference in the positioning of EGFP-expressing proteins were noted between the wildtype and the missense mutants (Fig. 1C). However, it was clear that the Cep290 constructs were recruited to the area of the ciliary base when compared to EGFP-vector transfected cells. The result is consistent with evidence presented by previous studies showing that CEP290 is localised at the proximal end of the ciliary axoneme^24,39^. Next, the ciliated fractions in starved NIH/3T3 cultures were assessed using antibodies against acetylated-tubulin to label the microtubule axoneme in primary cilium (Fig. 1D) and the length of cilia were measured (Fig. 1E). Although slight differences in both ciliated fraction and axoneme length were observed among the transfected cells, these changes were not statistically significant.

Regulation of primary cilium assembly and function is closely related to the entry and exit of the mitotic cell cycle process^5,40^. When cells exit the cell cycle and enter a quiescent state, the appearance of primary cilium is often observed^41^ and CEP290 have been proposed to play a crucial role in regulating this process^20,26^. Thus, the effects of Cep290 WT and mutants constructs on the occurrence and morphology of primary cilium in serum-stimulated cells were subsequently examined. In contrast to the serum-starved condition, there was a significant increase both in number of primary cilium-bearing cells (Fig. S1A) and in cilia axonemal length in serum-stimulated cultures overexpressing WT Cep290 (Fig. S1B). Interestingly, cells transfected with the R1747Q but not D665G variant had a similar fraction of cells bearing primary cilium and cilia length as the vector control. This data not only recapitulates the function of CEP290 as a potent cilia stabilisation factor, but also indicates that the R1747Q mutation alters the ability of CEP290 to execute this function.

Our data shows that the cilia stabilisation effect of overexpressed CEP290 is more pronounced in cycling rather than in quiescent cells. This observation is in agreement with previous findings in RPE-1 cells and might be related to a proposed mechanism in which an abundance of CEP290 protein molecules sequester the interaction partner CP110, which during serum-containing conditions inhibits CEP290, and thereby promote cilia formation^26^. In light of the finding that the R1747Q variant lacks the ability to promote cilia formation in proliferating cultures, it would be interesting to further examine if this mutation interferes with the binding to CP110. However, the following experiments were performed in serum-starved cultures to examine the molecular events in primary cilia under a more homogenous condition.

### Shh-signalling response is impaired in cells expressing the R1747Q mutant and leads to altered subcellular distribution of Smoothened

**Figure 2:**
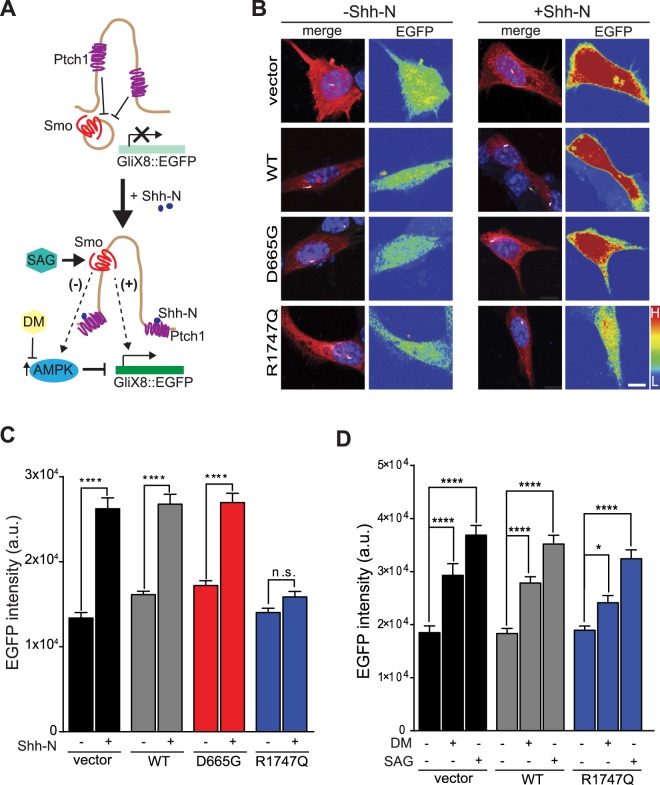
Overexpression of the R1747Q variant impairs the response of Shh stimulation but not signalling downstream of Smoothened. (**A**) Simplified illustration of Shh-N mediated activation of the GliX8::EGFP reporter. In absence of Shh-N, Ptch1 inhibits Smo from entering the cilium and Gli-dependent gene transcription of Shh target genes as well as the EGFP reporter is inactive. Upon Shh-N stimulation, Ptch1 exits the cilium and allows for Smo translocation to occur. Smo thereafter activates Gli which in turn leads to downstream EGFP expression (+). Alternatively, a negative feedback mechanism has been proposed in which the presence of ciliary Smo might trigger AMPK-mediated destabilisation of Gli and reduction of gene expression (−). However, by using DM to inhibit phosphorylation of Gli by AMPK, EGFP production can be restored. Transcription of the Shh pathway reporter plasmid can also be achieved by direct pharmacological activation of Smo using Smoothened Agonist (SAG). Cells were cotransfected with the Shh reporter construct GliX8::EGFP and RFP-vector or indicated mCherry-Cep290 plasmids, and thereafter starved for 24 hrs prior to 48 hrs of pharmacological treatment. (**B**) Representative images of cells treated with vehicle control (0.1% BSA in ddH_2_O) or recombinant Shh, Shh-N (6 μg/mL). Merged micrograph compilations show RFP or mCherry (red), acetylated tubulin (white) and DAPI staining (blue). Pseudo colour indicates EGFP fluorescence intensities in the cell soma (L = low intensity; H = high intensity). Scale bar = 10 μm. (**C**) Graph shows the mean fluorescence intensity of EGFP in the soma of transfected cells with and without Shh-N stimulation. Results were obtained from three independently performed experiments, n ≥ 113 cells per group. (**D**) Quantified EGFP mean intensity measured in the soma of fixed cells transfected and treated with either vehicle (sterile ddH_2_O), Dorsomorphin (DM, 1 μM) or Smoothened Agonist (SAG, 400 nM), n ≥ 49 cells per condition, three independent experiments. (**C**,**D**) Data are represented as mean ± SEM. *p < 0.05, ****p < 0.0001 compared to respective vehicle-treated control for each group, Mann-Whitney unpaired t-test in (**C**) or one-way ANOVA *post hoc* Holm-Sidak’s multiple comparisons test in (**D**).

The primary cilium contains a highly specialised and compartmentalised molecular environment. For example, the Sonic hedgehog (Shh) signalling machinery is selectively localised to the primary cilium^42^. To determine whether Shh signalling is altered in the Cep290 mutant NIH/3T3 cells, mCherry-tagged constructs were co-expressed with the GliX8::EGFP, a GliX8 (eight Gli-binding sites-containing promoter) Shh reporter construct modified to drive EGFP expression when Gli transcription is turned on upon activation of Shh signalling^43^ (Fig. 2A). Cells were thereafter stimulated with recombinant murine Shh peptide (Shh-N) or with vehicle (0.1% BSA in ddH_2_O) and activity-dependent EGFP expression was detected in the cell soma (Fig. 2B) and quantified (Fig. 2C). Cells transfected with vector control, WT and D665G CEP290, responded robustly to Shh stimulation, as shown by an increase in EGFP intensity (vector − 196 ± 10%; WT − 167 ± 6%; D665G − 158 ± 5%). However, cells transfected with the R1747Q variant displayed a modest (115 ± 3%) and non-significant Shh-dependent response.

**Figure 3:**
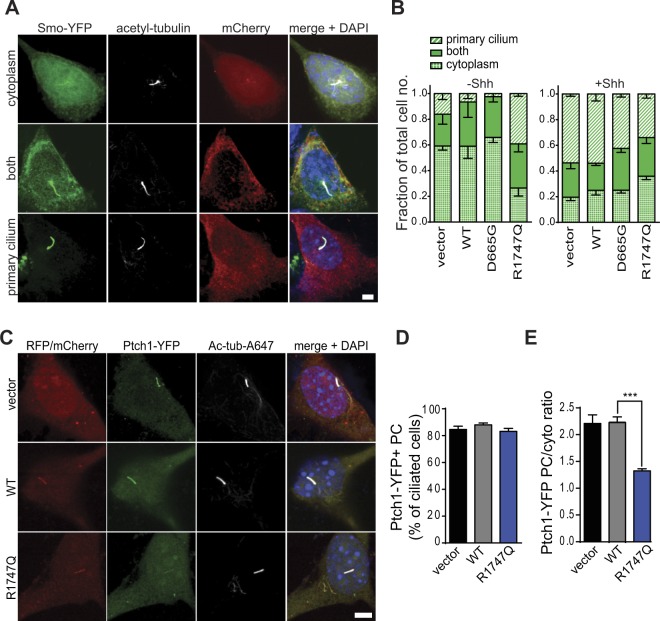
Smo distribution as well as Ptch1 ciliary enrichment are altered in cells overexpressing the R1747Q variant. (**A**) Micrographs show non-stimulated cells representing the three different categories of Smo localisation observed in NIH/3T3; only cytoplasmic compartments (cytoplasm), both cytoplasm and primary cilium (both), and only primary cilium (primary cilium). To determine the localisation of Smoothened, NIH/3T3 cultures were cotransfected with Smo-YFP and mCherry-Cep290 constructs and starved for 24 hrs. Scale bar = 5 μm. (**B**) Quantification of the different Smo localisation pattern in cells expressing indicated constructs and treated with vehicle or Shh-N for 1 hr prior to immunostaining procedures. n ≥ 50 cells per group and treatment, three independent experiments. (**C**) Images of starved, non-stimulated NIH/3T3 cells cotransfected with Ptch1-YFP and indicated RFP/mCherry constructs demonstrating typical ciliary enrichment of Ptch1-YFP. Scale bar = 5 μm. (**D**) Percentages of cells with Ptch1-YFP positive (Ptch1-YFP+) primary cilia (PC) were quantified for each condition (n ≥ 201, four independent experiments). (**E**) Quantification of Ptch1-YFP enrichment in PC of transfected cells calculated as the ratio between the YFP mean fluorescence intensity within a PC-ROI and an equally sized reference cytosolic ROI (n ≥ 69 cells per condition, four individual experiments). (**B**,**D**,**E**) Data are represented as mean ± SEM. ***p < 0.001, one-way ANOVA *post hoc* Holm-Sidak’s multiple comparisons test.

A key step in the Shh signal transduction scheme is the Patched1 (Ptch1) Shh receptor-coordinated translocation of Smoothened (Smo) from the cytosol into the primary cilium, which ultimately results in activation of Gli-dependent gene transcription^44,45^. To further investigate the mechanism for the muted Shh response in R1747Q mutant cells, the localisation of Smo in Shh-N stimulated, and unstimulated cells was examined. NIH/3T3 cells were co-transfected with Smo-YFP and either vector or mCherry-Cep290 constructs. We categorised the cellular distribution patterns of Smo-YFP into three distinct categories: only cytoplasmic compartments (cytoplasm), both cytoplasm and primary cilium (both), and only primary cilium (primary cilium) (Fig. 3A). The fractions of cells displaying these different Smo distribution patterns were quantified (Fig. 3B). In the unstimulated condition, Smo was preferentially localised to the cytosol of cells transfected with vector, WT Cep290 and D665G variant (cytosol: vector − 0.59 ± 0.03, WT − 0.59 ± 0.10, D665G − 0.69 ± 0.04; both: vector − 0.25 ± 0.08, WT − 0.34 ± 0.12, D665G − 0.32 ± 0.04; primary cilium: vector − 0.16 ± 0.05, WT − 0.07 ± 0.04, D665G − 0.02 ± 0.02). However, Smo was already preferentially located in the primary cilium in cells with R1747Q mutant (cytosol: 0.26 ± 0.06; both: 0.34 ± 0.06; primary cilium: 0.39 ± 0.02). Shh-N stimulation caused translocation of Smo into primary cilia of vector-, WT- and D665G-tansfected cells (cytosol: vector − 0.20 ± 0.03, WT − 0.25 ± 0.04, D665G − 0.25 ± 0.02; both: vector − 0.27 ± 0.05, WT − 0.21 ± 0.02, D665G − 0.33 ± 0.03; primary cilium: vector − 0.54 ± 0.02, WT − 0.54 ± 0.06, D665G − 0.42 ± 0.02). The Smo-YFP distribution pattern displayed by the cells expressing the R1747Q variant was not significantly changed by the addition of Shh-N (cytosol: 0.36 ± 0.03; both: 0.30 ± 0.05; primary cilium: 0.34 ± 0.03). This data suggests that the altered localisation pattern of Smo may underlie the defective Shh response in R1747Q mutant cells. The failed Shh stimulation-induced recruitment of Smo to the ciliary compartment further indicates that the mechanism regulating Smo redistribution is disrupted.

Since Smo ciliary localisation is restricted by the presence of Ptch1 in primary cilium when Shh is absent, the localisation of Ptch1 in the R1747Q mutant cells in the unstimulated condition was examined (Fig. 3C). Analysis of the frequency of ciliary enrichment of Ptch1-YFP proteins revealed although there were no differences in the percentages of cells containing Ptch1-YFP positive cilia (vector − 85 ± 2%; WT − 88 ± 1%; R1747Q − 83 ± 2%) (Fig. 3D), the R1747Q mutant cells displayed a significant decrease in the ciliary enrichment of Ptch1 compared to vector- and WT-transfected cells (YFP mean intensity PC/cyto ratio: vector − 2.21 ± 0.16; WT − 2.50 ± 0.10; R1747Q − 1.32 ± 0.04) (Fig. 3E). This indicates that the Smo mislocalisation phenotype associated with the R1747Q mutation could be related to an upstream defect in regulation of Ptch1 ciliary confinement.

The absence of Shh-N stimulated Gli transcriptional activation despite the increase of primary cilium localization of Smo receptors in R1747Q mutant cells suggests that the Smo-mediated signalling pathway is impaired. In a recent study, it was hypothesised that Smo translocation to primary cilia might lead to activation of AMP-activated protein kinase (AMPK)^46^. This, in turn, could trigger a downstream negative feedback of Shh-signalling due to selective inhibitory phosphorylation of Gli1, as previously demonstrated^47^. The relatively high concentration of Smo in cilia of R1747Q transfected cells could potentially thereby promote a state of increased AMPK phosphorylation and subsequent destabilisation of Gli1, thus inhibiting Gli1-dependent expression of the GliX8::EGFP reporter (Fig. 2A). To test whether increased AMPK activity might interfere with the ability of R1747Q-expressing cells to respond to Shh-N, pharmacological inhibition of AMPK was performed using Dorsomorphin (DM), a compound previously shown to effectively attenuate AMPK activity^48^. By blocking this negative feedback control mechanism of Shh signalling, Gli-mediated expression of the EGFP reporter could be restored in R1747Q mutant cells to levels comparable to vector and WT Cep290 (vector − 158 ± 12%; WT − 152 ± 6%; R1747Q − 127 ± 8%) (Fig. 2D). This data suggests that the signalling cascade downstream of Smo is still functional in R1747Q mutant cells. The reason for the muted Shh response despite an increase of Smo localization in primary cilia may be the inactive state of the receptor. It has been shown that the initiation of Shh signalling requires not only the localisation of Smo in the primary cilia, but also the activation of the receptor^49^. Consistent with this hypothesis, cells were stimulated with SAG, a small molecule agonist acting directly on Smo which is shown to activate the receptor leading to downstream Gli gene expression and Smo translocation to cilia^50^. SAG stimulation robustly activated Gli reporter plasmid expression of R1747Q expressing in cells at a level similar to control and WT Cep290 (vector − 199 ± 10%; WT − 192 ± 9%; R1747Q − 171 ± 9%) (Fig. 3D). However, similar to Shh-N stimulation, treatment with SAG did not change the Smo localisation pattern in cells transfected with the R1747Q variant (Fig. S2). Therefore, the failure of dynamic organisation and localisation of receptors responsible for Shh pathway activation appears to be the key mechanism underlying the muted Shh response in R1747Q mutant cells.

### CEP290-R1747Q missense mutation affects mobility of ciliary membrane-associated proteins

**Figure 4:**
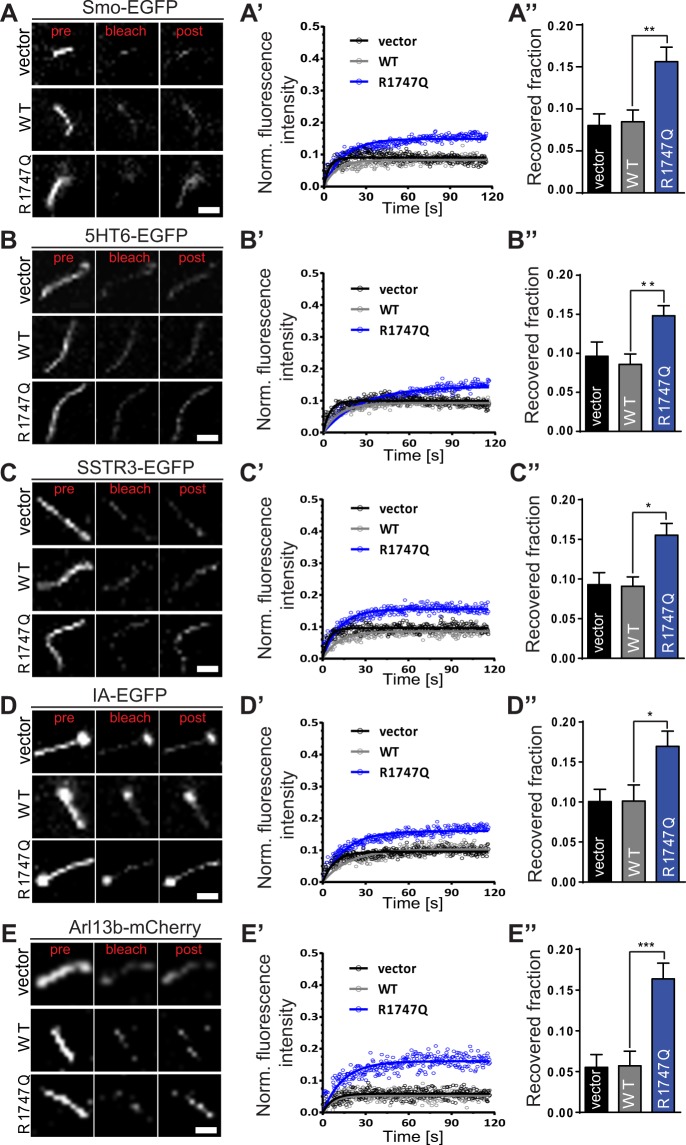
FRAP analysis reveals that the R1747Q missense mutation affects the mobility of ciliary molecules. Cells were cotransfected with indicated mCherry- or EGFP-tagged ciliary proteins and vector or mCherry/EGFP-Cep290 constructs and starved for 24 hrs prior to FRAP experiments. Representative images show changes in fluorescence intensity prior (pre; t = −3.146 s), at (bleach; t = 0.000) and after (post; t = 115.191 s) photobleaching of (**A**) Smo-EGFP, (**B**) 5HT6-EGFP, (**C**) SSTR3-EGFP, (**D**) IA-EGFP, and (**E**) Arl13b-mCherry in primary cilium. Scale bar = 2 μm. (A’–E’) Fluorescence recovery of indicated bleached proteins are plotted as recovery curves in each transfected condition. Data was corrected for background fluorescence and for FRAP-unrelated bleaching effects using an unbleached ROI and thereafter normalised to the fluorescence intensity immediately before bleaching. (A”–E”) Recovered fraction after bleaching was obtained from the plateau of the recovery curves. n ≥ 18 for Smo-EGFP, n ≥ 32 for 5HT6-EGFP, n ≥ 29 for SSTR3-EGFP, n ≥ 23 for IA-EGFP, and n ≥ 33 for Arl13B-mCherry. n = cells per category, three independent experiments. Data are represented as mean ± SEM. *p < 0.05, **p < 0.01, ***p < 0.001 compared to vector, one-way ANOVA *post hoc* Holm-Sidak’s multiple comparisons test.

Proteomic studies have shown that the primary cilium is a specialised cellular compartment with a highly defined, unique and restrictive molecular milieu despite its closeness to the cytoplasmic vicinity^46,51,52^. Hence, free diffusion of proteins between the cytosol and the primary cilium is unlikely to occur. Ultrastructural analysis by electron microscopy and super resolution microscopy has revealed that CEP290 resides at Y-link junctions of the ciliary transition zone and stabilises the linkage of axoneme microtubules to the ciliary membrane^16,21,22^. For that reason, a ciliary ‘gatekeeper’ function has been postulated for CEP290 to maintain the integrity of the ciliary diffusion barrier and regulate the entry and exit of ciliary proteins^16,23–25^. Likewise, this function of CEP290 is critical for positioning the molecular components that are necessary for correct processing of Shh signalling in the primary cilium. Thus, whether the lack of Shh response and the mislocalisation of Smo observed in cells expressing the R1747Q missense mutation could be connected to defects in the ciliary gating ability of Cep290 was investigated. To that end, live-cell imaging combined with fluorescence recovery after photobleaching (FRAP) analysis was performed to determine the dynamics movements of primary cilium receptors and proteins (Fig. 4).

First, to confirm that the changed Smo localisation pattern observed in R1747Q expressing cells is connected to a disruption in ciliary gating function, the fluorescence intensity recovery ability of Smo-EGFP molecules in cilia was determined (Fig. S3A). Indeed, in support with the results from the Smo distribution analysis, the Cep290-R1747Q expressing cells displayed an increase in the fraction of Smo-EGFP molecules able to populate the cilium area (Recovered fraction) after irreversible photobleaching when compared to vector and WT Cep290 (vector − 8.0 ± 1.4%; WT − 8.5 ± 1.4%; R1747Q − 15.6 ± 1.7%) (Fig. 4A-A”). To further assess whether the gating defect of R1747Q mutant affects other ciliary transmembrane proteins, the fluorescence recovery of two EGFP-fused primary cilium-targeted receptors: 5-hydroxytryptamine receptor 6 (5HT6) and somatostatin receptor 3 (SSTR3) (Fig. S3B,C) was examined. Consistent with the FRAP recordings performed with Smo-EGFP, an increase in mobility of these two ciliary receptors was observed in cells expressing the R1747Q variant (5HT6: vector − 9.6 ± 1.8%; WT − 8.6 ± 1.3%; R1747Q − 14.8 ± 1.7%; SSTR3: vector − 9.2 ± 1.5%; WT − 9.1 ± 1.2%; R1747Q − 15.5 ± 1.5%) (Fig. 4B-C”).

It has been demonstrated that the loss of ADP ribosylation factor like GTPase 13B (Arl13b) results in displacement of key components of the Shh signalling machinery, such as Smo, and causes abnormal Shh signalling^53,54^. To address whether the mislocalisation of Smo in R1747Q mutant cells could be linked to a potential dysregulation of Arl13b ciliary confinement, FRAP analysis was performed and Arl13b movement between the cytosol and the primary cilium was recorded (Fig. S3E). Arl13b-mCherry significantly increased recovery in cells expressing R1747Q mutant (RF: vector − 5.5 ± 1.6%; WT − 5.7 ± 1.8%; R1747Q − 16.4 ± 1.9%) (Fig. 4E,E”). Additionally, an artificially engineered transmembrane construct, β_1_-integrin-Arl13b (IA)^55^ was examined for its movement (RF: vector − 10.1 ± 1.5%; WT − 10.1 ± 2.0%; R1747Q − 17.0 ± 1.9%) (Fig. 4D-D”; S3D).

In conclusion, these data strongly suggest that the R1747Q-dependent ciliary gate deficiency phenotype appears non-discriminative in nature and not restricted to a specific ciliary protein. The results support the notion that, in addition to the abnormal ciliary localisation of Smo, Cep290 proteins carrying the R1747Q mutation exhibit a diminished ability to execute its gatekeeper function and a failure to regulate the trafficking of other ciliary membrane-associated receptors.

### Patient-derived iPSCs harbouring the R1746Q missense mutation in CEP290 exhibit deficits in stability, morphology and gating of the primary cilium as well as a reduced response to Shh stimulation

**Figure 5:**
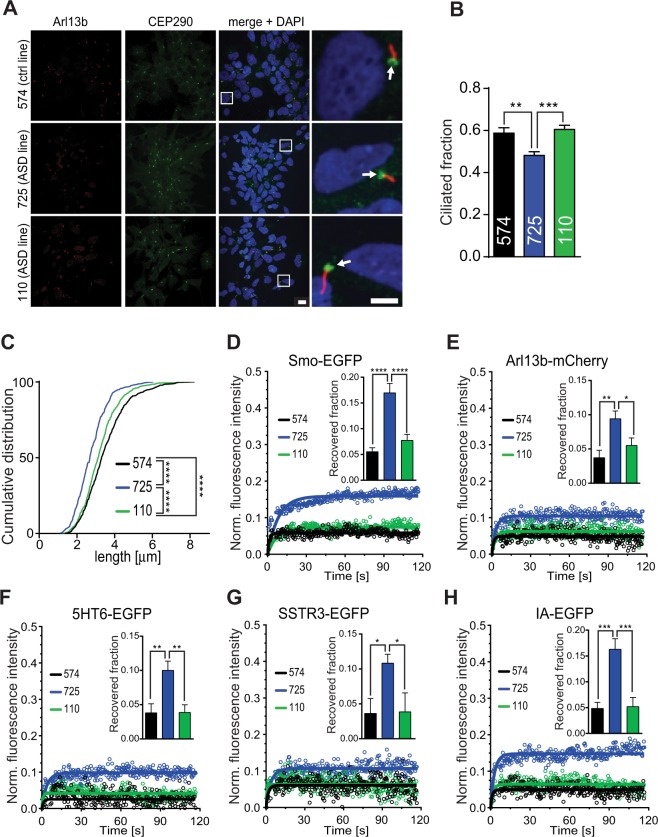
A patient-derived iPSC line containing the CEP290-R1746Q variant (#725) exhibits altered cilia morphology and function as well as diminished response to Shh-N stimulation. (**A**) Maximum intensity projections of confocal image z-stacks of iPSC cultures typically used for assessment of ciliated cell fractions and cilium length measurements. Immunostaining was used for detection of ciliary axonemes (Arl13b) and for subcellular localisation of CEP290. White squares indicate zoomed-in regions. White arrows point to localisation of CEP290 at the base of cilia. Scale bars = 10 μm and 5 μm. (**B**) Quantification of cilia-bearing cell fractions in iPSC cultures, n = 36 z-stacks/cell line; three independent experiments. (**C**) Cumulative frequency distribution of cilia lengths in iPSC cultures, n = 748 (#574 line), 539 (#725 line), 822 (#110 line); three separate experiments. ****p < 0.0001, Kolmogorov-Smirnov t-test. (**D**–**H**) FRAP curves generated as described in Fig. 4. Bar graphs representing the recovered fraction for each fluorescent protein analysed are inlayed in respective curve plots. n ≥ 23 for Smo-EGFP, n ≥ 25 for Arl13B-mCherry, n ≥ 15 for 5HT6-EGFP, n ≥ 18 for SSTR3-EGFP, and n ≥ 16 for IA-EGFP. n = cells per category, three independent experiments. Data are represented as mean ± SEM, *p < 0.05, **p < 0.01, ***p < 0.001, ****p < 0.0001, one-way ANOVA *post hoc* Holm-Sidak’s multiple comparisons test.

Mutations in CEP290 were recently found to be correlated with alterations in the average length of cilia in populations of JS patient-derived fibroblasts and primary renal epithelial cells^56,57^. Here, the primary cilium phenotype in human peripheral blood mononuclear cell (PBMC)-derived induced pluripotent stem cells (iPSCs) from an autistic individual carrying the CEP290-R1746Q variant (iPSC line #725) was examined. Consistent with the CEP290 overexpression data in NIH/3T3, the subcellular localisation of CEP290 at the ciliary base was not affected in the #725 iPSC line carrying the R1746Q mutation (Fig. 5A). However, both the fraction of cilia bearing cells (Fig. 5B) as well as axoneme length (Fig. 5C) were significantly reduced in the #725 iPSC line when compared to an iPSC control line (#574) and a cell line from an ASD patient lacking the CEP290 mutation (#110). A similar finding was described previously when comparing cultured wildtype mouse fibroblasts to fibroblasts from the *rd16* mouse, which expresses a microtubule-binding deficient variant of CEP290^26^. Interestingly, spontaneous synaptic network-level spiking activity, single cell calcium-transients, and the migration of neurites in neurones derived from iPSCs created from individual #725 were all significantly lower than controls^58^. Taken together, this suggests that the CEP290 mutation in this line not only affects the ciliary phenotype during stem cell maintenance, but may furthermore play a physiological role past the iPSC stage.

Unlike most of the CEP290 mutations that result in truncated or non-coding protein, the R1746Q variant encodes a full-length protein. With most of CEP290 variants previously examined; the lower the expression level of the protein, the longer the primary cilium axoneme^56,57^. Intriguingly, we observed a shorter cilia length in cells expressing R1746Q variant. Although we cannot measure the protein level of the CEP290-R1746Q in human iPSCs due to the limitation of the antibody, which does not distinguish the mutant versus the wild type protein, the mouse R1747Q variant is present at a similar level to that of the wild type when exogenously expressed in NIH3T3 cells (Fig. 1B). This suggests that in human cells the R1746Q variant likely is present at a comparable concentration to the wild type protein. Thus, in this case, our R1746Q-iPSCs contain a relatively high CEP290 protein level compared to other variants previously reported and could result in shorter primary cilia length as observed.

Next, to examine if the defective ciliary gating capacity seen in the Cep290-R1747Q-transfected NIH/3T3 cells is a feature shared by the #725 iPSC line, the FRAP-based analysis of ciliary protein mobility approach was employed (Fig. 5D–H). Cultures were transfected with the same battery of proteins previously examined in NIH/3T3. Similar to what was observed in the mouse cell line, iPSCs carrying the R1746Q mutation showed considerably increased fluorescence recovery in the bleached cilium region compared to iPSCs expressing the normal CEP290 variant (Smo: 574 − 5.5 ± 0.8%; 725 − 17.0 ± 1.8%; 110 − 7.7 ± 1.2%; Arl13b: 574 − 3.7 ± 1.1%; 725 − 9.4 ± 1.1%; 110 − 5.5 ± 1.1%; 5HT6: 574 − 3.8 ± 1.3%; 725 − 10.0 ± 1.3%; 110 − 3.9 ± 1.1%; SSTR3: 574 − 3.6 ± 2.1%; 725 − 11.0 ± 1.3%; 110 − 3.9 ± 2.7%; IA: 574 − 4.8 ± 1.2%; 725 − 16.3 ± 2.0%; 110 − 5.2 ± 1.8%). Taken together, this data suggests that the expression of this rare CEP290 variant results in abnormal cellular phenotypes which can be detected in both mouse and human cells. This translational approach could provide a platform for further investigations to understand the potential mechanism disrupted in the neuropathological state.

### CEP290-R1747Q fails to regulate the proliferation of granule cell progenitors *in vivo*

Brain abnormalities in autism and CEP290-related ciliopathies are most commonly found in the cerebellum and have been proposed to be linked to defects in the expansion of the cerebellar granule cell population during postnatal development^59,60^. As this process is Shh-dependent^61^, it raises the question of whether the R1746Q variant could interfere in the process of cerebellar development.

**Figure 6:**
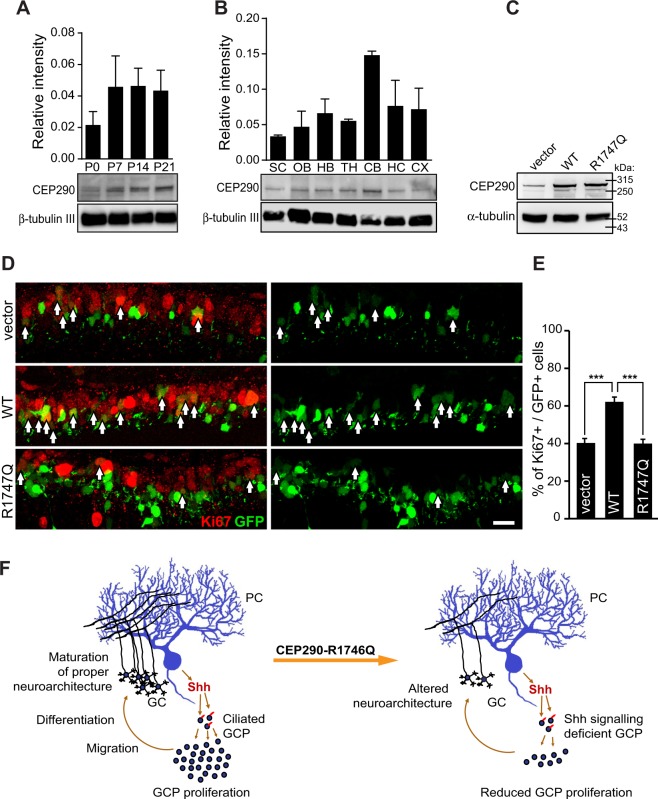
Cep290-R1747Q fails to maintain GCPs in the proliferative state during cerebellar development. Immunoblotting analysis of Cep290 protein expression in mouse whole brain lysate harvested (**A**) at indicated postnatal days (P) or (**B**) in indicated brain regions harvested from P14 pups. Bar graphs show Cep290 protein abundance quantified as intensity of Cep290 bands relative to reference protein (β-tubulin III) (three independent experiments). See also Fig. S4D–G for full blots. SC = spinal cord, OB = olfactory bulb, HB = hind brain, TH = thalamus, CB = cerebellum, HC = hippocampus, CX = cortex. (**C**) HEK293T cells transfected with pCIG2, pCIG2-Cep290 or pCIG2-Cep290-R1747Q constructs. Both pCIG2-Cep290 and pCIG2-Cep290-R1747Q showed elevated Cep290 expression at a similar level. (**D**) Immunostaining of the cell cycle marker Ki67 in mouse cerebellar slices electroporated with pCIG2, pCIG2-Cep290 or pCIG2-Cep290-R1747Q constructs at P6. Ki67+ (red) cell among electroporated cells, which also express EGFP (green) are indicated with arrows. Scale bar = 20 μm. (**E**) The bar graph shows an increased percentage of Ki67+ GCPs in cerebella electroporated with pCIG2-Cep290 (n = 4 animals) but not pCIG2-Cep290-R1747Q (n = 6 animals) compared to the pCIG2-vector control (n = 7 animals). ***p < 0.001, One-way ANOVA. Data are represented as mean ± SEM. (**F**) Working model of CEP290-R1746Q mediated alteration in the neurodevelopment of the cerebellum. In the neurotypical brain, Shh secreted by Purkinje cells (PC) are sensed by ciliated GCPs and results in expansion of the progenitor pool and the consequent establishment of the proper cerebellar neurocircuitry. However, GCPs expressing the dominant negative CEP290-R1746Q variant are defective in their response to the Shh morphogen thus resulting in a diminished GCP population and consequent neuroarchitectural alterations.

First, the spatiotemporal expression of Cep290 in the mouse brain was determined. Western blot analysis of Cep290 from mouse whole brains isolated from different postnatal days (P) showed that the expression of Cep290 increased and reached a plateau at P7, around the time of granule cell proliferation burst (Fig. 6A). Cep290 expression also showed the most abundance in the cerebellum compared to other brain regions at P14 (Fig. 6B).

To achieve a better expression *in vivo*, Cep290 constructs were re-engineered into the pCIG2 vector. Similar to the expression profiles in NIH/3T3 cells, transfection of pCIG2-Cep290 and pCIG2-Cep290-R1747Q in HEK293T cells resulted in a comparable expression of the CEP290 protein as the pCIG2 control (Fig. 6C). These constructs were then electroporated into cerebellar GCPs in mice at P6. Two days later, electroporated cerebella were fixed, sectioned, and immunostained with the cell cycle marker Ki67, which labels cells during proliferation (Fig. 6D). CEP290 overexpression in GCPs led to a higher percentage of Ki67+ cells compared to the control cerebella electroporated with the empty vector (pCIG2-Cep290: 62.2 ± 2.6%, pCIG2: 40.3 ± 2.3%). As the postnatal expansion of the GCP population requires a functional Shh signalling machinery coordinated by the primary cilium, the proliferative ability of these cells is thus dependent on the presence of intact cilia structures^62,63^. The additive effect on GCP proliferation by Cep290-WT expression is likely due to an escalation in the number of ciliated cells, as suggested by the phenotype shown in NIH3T3 cells (Fig. S1A). The increased fraction of cells with primary cilium can be explained by the cilia stabilisation function of Cep290, a feature which appears lost in the Cep290-R1747Q mutant and results in no additional elevation of the number of cilium-bearing cells above that observed in controls. In line with that finding, the defective Shh signalling phenotype associated with the Cep290-R1747Q variant should prevent the granule progenitor cells from responding to the Shh-dependent proliferation induction. Consequently, in GCPs, the expression of the Cep290-R1747Q mutant did not generate a higher percentage of Ki67+ cells compared to the control (Ki67+: 40 ± 2.7%) and thus lacks the WT protein effect on proliferation (Fig. 6E). Since proliferation of GCPs is dependent on Shh signals produced by Purkinje neurones, the presence of the Cep290 R1747Q missense mutation that results in defective Shh-signalling machinery may contribute to further failure in circuit formation and altered neuroarchitectural development of the cerebellum (Fig. 6F).

## Conclusion

The present study shows that the mouse analogue of the autism-associated R1746Q mutation in CEP290 has a dominant negative effect on the regulatory function of CEP290 to coordinate cell proliferation and to stabilise the molecular integrity of the primary cilium. Consequently, expression of the R1746Q mutation ultimately results in the displacement of several ciliary transmembrane proteins, a feature which is connected to the finding that an impaired Shh-N stimulation response is observed in cells carrying this CEP290 variant. In a neurophysiological context, neurotypical development of the cerebellum requires cell communication by the Shh morphogen to trigger proliferation, differentiation and migration of the cerebellar granule cell progenitors^61,64,65^. Since alterations in the cerebellar neuroarchitecture have been identified in individuals with autism^66^, the results of this study warrant further investigation into the potential contribution of primary cilium-linked cell signalling events in ASD. Therefore, insights into the cellular mechanisms by which the primary cilium participates in neurodevelopment could provide valuable avenues in the pursuit to understand neurobiological disorders.

## Methods

### NIH/3T3 cell culture

NIH/3T3 cells (ATCC) were maintained at 37 °C in a humidified 5% C02 incubator (Thermo Fisher) in full culture medium (DMEM supplemented with 10% iron-fortified FCS, 1% Penicillin/Streptomycin and 1% L-glutamine) and passaged every 3–4 days or before cells reached more than 80–90% confluence. Cells were not allowed to reach a passage number higher than 15. Unless otherwise specified, cells were seeded at the following densities: 6-well plates and 35 mm dishes–750,000 cells/well or dish; 24-well plates–150,000 cells/well; 96-well plates–10,000 cells/well. All cell culture chemicals were purchased from Life Technologies and all cell culture plastics were from Sigma, if not stated otherwise.

### Human iPSC (hiPSC) culture

PBMC-derived hiPSCs were generated as described previously^58,67,68^ and stem cell colonies were propagated in complete culture medium containing mTeSR^TM^1 basal medium (Stemcell Technologies) supplemented with mTeSR^TM^1 5X supplement (Stemcell Technologies), 1 μM Thiazovivin (Stemgent), 10 μM CHIR99021 (Stemgent), 1 μM PD0325901 (Stemgent) and 40 ng/ml basic FGF (Stemgent) on a layer of mitotically inactive CF-1 γ-irradiated MEF cells (Life Technologies). Medium was replenished every day by 50% exchange and cells were passaged every 7 days. For establishment of cultures undifferentiated iPSC colonies were selected and thereafter dissociated in Accutase for 7 mins at 37 °C. After removal of Accutase and gentle trituration in passaging medium (complete culture medium containing 20 ng/ml basic FGF and 10 μM Y27632 (Stemgent), cell suspension was pre-plated in gelatin (0.1% in PBS)-coated 100 mm dishes twice for 45 mins in order to remove residual MEF feeder cells. Subsequently, iPSCs were seeded onto either HCl-etched and 83.3 μg/ml Matrigel (BD)-coated 12 mm glass coverslips (Carolina Biologicals) (100,000 cells/glass coverslip) or in Matrigel-coated 12-well plates (150,000 cells/well) or 35 mm glass bottom dishes (300,000 cells/dish). 18–24 hrs after plating passaging medium was changed to complete culture medium and cells were grown for either 48 or 24 hrs prior to live cell imaging or fixation and immunocytochemistry procedures respectively.

### Animals

C57BL/6 mice were purchased from the animal facility of the University of Maryland School of Medicine and housed and cared for by the AAALAC accredited program of the University of Maryland School of Medicine. ICR mice were maintained in the animal facility of National Yang-Ming University. All experiments were reviewed and approved by the Institutional Care and Use Committees (IACUC) of the University of Maryland School of Medicine, the Hussman Institute for Autism, and National Yang-Ming University. All experimental procedures were performed in accordance with the guidelines and regulations of each institution.

### Immunocytochemistry

Sterile 12 mm glass cover slips placed in 24-well plates were coated with poly-D-lysine (20 μg/ml PDL in 20 mM HEPES). Cells were transfected and plated as described earlier. Cultures were subjected to stimulation or starving procedures described in subsequent sections. Cover slips were fixed in ice-cold 4% PFA, washed with PBS and subsequently incubated with blocking solution (1% BSA, 1% normal donkey serum and 0.3% Triton X-100 in PBS). During all procedures, the plate was covered to minimise loss of fluorescence signal. Primary and secondary antibodies were diluted in blocking solution and 2-Phenylindole-4‘,6-dicarboxamidine dihydrohydrochloride (DAPI, Invitrogen) and Hoeschst-33342 (Life Technologies) final concentrations were 300 nM and 5 μg/ml respectively in PBS. Antibodies used were rat anti-RFP (clone 5F8, antibodies-online Inc.), mouse anti-acetylated tubulin (clone 6-11B-1, Sigma-Aldrich), rabbit anti-GFP (Covalab SAS), mouse anti-Arl13b (clone N295B/66, NeuroMab), rabbit anti-CEP290 (Bethyl laboratories Inc.), Alexa Fluor® 488 donkey anti-rabbit IgG, Alexa Fluor® 594 donkey anti-rat IgG and Alexa Fluor® 647 donkey anti-mouse IgG (all from Life Technologies). Cover slips were mounted on glass slides using ProLong Diamond antifade mountant (Life Technologies).

### Morphometric analysis of primary cilia

NIH/3T3 cells were either starved for 24 hrs using starving medium (culture medium w/o FCS) to inhibit proliferation or cultured in full culture medium to promote mitosis. hiPSCs were maintained in complete culture medium. After immunocytochemistry, fluorescence imaging was performed on an upright Zeiss 780 confocal LSM using a Plan-Apochromat 63x/1.4 oil objective. NIH/3T3 cilia-bearing fractions were determined by counting the number of cells with a single, prominent acetylated tubulin-positive protrusion among the cells expressing EGFP constructs and normalising to the total number of cells with EGFP signal. Fractions of cilia-bearing hiPSCs were determined by counting the number of cells exhibiting a single Arl13b-positive axoneme. For assessment of cilia length, Z-stacks of 6–10 μm depth were acquired, and measurements were done in ZEN 2012 SP1 software either on maximum intensity projections or, whenever necessary due to the position of the cilium, using the ortho-tool to assess the length in the Z-stack cross section projection. Samples were blinded before counting/measurement of cilia. All raw data was processed in Excel Office Professional 2013 and exported to GraphPad for further statistical analysis.

### Glix8::EGFP Shh pathway response reporter assay

Transfection, plating and starving of cells were done as described in the supplementary information. Cells were treated for 48 hrs with either vehicle (0.1% BSA in ddH_2_O), 6 μg/mL recombinant murine Shh-N (Pepro Tech) reconstituted in vehicle solution, 1 μM Dorsomorphin (Enzo Life Sciences), or 400 nM Smoothened Agonist (Alfa Aesar). Fixed and immunostained samples were imaged with an upright Zeiss LSM 780 confocal and an EC Plan-Neofluar 40x/1.3 oil objective. Only cells with comparable RFP/mCherry fluorescence intensity were used for measurements of EGFP fluorescence intensity within the soma. The mean intensity of EGFP was quantified by drawing a free-hand region of interest (ROI) around the cell soma using ZEN 2012 SP1 software. All samples were blinded before imaging and quantification.

### Evaluation of Smo-YFP and Ptch1-YFP distribution

Cells were plated on PDL-coated cover glass, transfected and starved as described earlier. Smo-YFP distribution assessment was done in cells treated for 2 hrs with vehicle or Shh-N (6 μg/ml) prior to fixation and cells were counted and grouped into three distinct categories of localisation pattern: solely cytoplasmic (cyto), a mixed distribution of cytoplasmic and ciliary (both), and restricted ciliary localisation (primary cilium). The presence and enrichment of Ptch1-YFP in primary cilium were determined by normalising the mean intensity of the YFP signal within an ROI matching the cilia to an ROI of equal size placed in the cytosol. Only cells displaying similar expression levels of Cep290/vector proteins were analysed. All samples were blinded for quantification. Data was compiled in Excel 2013 and statistical analysis was performed in GraphPad.

### Fluorescence recovery after photobleaching (FRAP) and live-cell imaging

Cells were plated on either PDL-coated (NIH/3T3) or Matrigel-coated (iPSCs) 35 mm glass-bottom dishes (Matsunami Glass USA Corporation). Live-cell imaging using an inverted Zeiss LSM 510 confocal microscope was performed 48 hrs after transfection and 24 hrs of starvation (for NIH/3T3 cells only). Fifteen minutes prior to imaging, the cell starving medium was replaced with Culture External Base containing 2 mM MgCl_2_, 2 mM CaCl_2_, 150 mM NaCl, 2.5 mM KCl, 10 mM glucose, and 10 mM NaHEPES. FRAP experiments were executed at room temperature using a Plan-Apochromat 40x/1.1 water objective. Images were acquired every 393 ms for a total of 300 cycles. An elliptical bleach ROI was tightly placed over the ciliary axoneme and bleaching of fluorescently tagged ciliary proteins was performed after 10 cycles using the 488 nm (EGFP) or 561 nm (mCherry) laser at maximal excitation power with 40 iterations. Background fluorescence (F_bg_) was determined in an area outside the cell using an ROI size equal to the bleach ROI. The fluorescence (F_corr_) of a same size ROI in an unbleached area with similar initial fluorescence as the bleach ROI was also measured to correct the potential FRAP-unrelated bleaching effects. Bleach ROI fluorescence intensity data (F_bleach_) was normalised using the equation F_norm_ = (F_bleach_ − F_bg_)/(F_corr_ − F_bg_). Fluorescence recovery (mobile fraction) over time (F(t)_FRAP_) was calculated as follows: F(t)_FRAP_ = (F_post_ − F(0))/(F_pre_ − F(0)), where F(0) is the normalised intensity directly after bleaching, F_post_ is the intensities at any time point after F(0) and F_pre_ is the initial intensity before bleaching. Recovery curves were analysed by a one-site association nonlinear curve fitting and recovered fraction (RF) was determined as the average of the recovery curve plateau values F(111–115). All imaging was performed at the Confocal Microscopy Core Facility at the University of Maryland School of Medicine.

### *In vivo* cerebellar electroporation

*In vivo* cerebellar electroporation was performed as described previously with some modifications^69^. Briefly, after anaesthesia by ice, a tiny hole in the skull was made above the cerebellum of ICR mice at P6. 1 μl of DNA at 3–5 μg/μl with 0.1% fast green were injected into cerebellar primary fissure by a 33-gauge needle. After injection, the 33-gauge needle served as the anode while a modified tweezer-type electrode connected to a cathode was placed on the occipital region. Six electric pulses at 70 V for 50 ms duration and 150 ms intervals was delivered by pulse generator (ECM 830, Harvard Apparatus). After electroporation, the wound was stitched up with a thread and then the post-operative mouse pups were re-warmed by placing on a heating pad. To harvest the cerebellum, mice were anaesthetised for 5–10 minutes on ice followed by transcardial perfusion of PBS and 4% paraformaldehyde (PFA). The cerebellum was further fixed by immersion in 4% PFA overnight after perfusion.

### Immunofluorescence staining of cerebellar slices

The cerebellum was sagitally sectioned by Vibrotome at 100 μm thickness. Slices were permeabilised by PBS-T (0.2% Triton-X 100 in PBS) for 30 minutes and blocked in blocking buffer (10% NGS and 5% BSA in PBS-T buffer) for 1 hr at room temperature. Slices were incubated with primary antibodies (Ki67, Millipore, 1:500) diluted in blocking buffer (5% NGS and 5% BSA in the PBS-T buffer) at 4 °C for two nights. Secondary antibodies were applied for 2 hrs at room temperature after incubated of primary antibodies. Finally, slices were stained with 0.5 μg/ml DAPI (Invitrogen) for 1.5 hrs. After sealing the slices, mounting buffer (VECTASHIELD ® Mounting Medium Media) were added into slices to maintain the fluorescence.

## Electronic supplementary material

Supplementary Information

## Data Availability

The data that support the findings of this study are available from the corresponding author on reasonable request.
