# Supplementary material for: A rare human CEP290 variant disrupts the molecular integrity of the primary cilium and impairs Sonic Hedgehog machinery
Source: Sci Rep. 2018 Nov 26;8:17335. doi: 10.1038/s41598-018-35614-x (PMC6255789; doi:10.1038/s41598-018-35614-x)
Supplement: Supplementary file 1 — Supplementary Information [file 41598_2018_35614_MOESM1_ESM.pdf]

Supplementary Information

Supplemental Methods and Figures

A rare human CEP290 variant disrupts the molecular integrity of the primary cilium and impairs Sonic Hedgehog machinery

Authors/Affiliations:

Michaela B.C. Kilander[1], Chun-Hung Wang[2], Chia-Hsiang Chang[2,3], Jonathan E. Nestor[1], Kevin Herold[5], Jin-Wu Tsai [2,4], Michael W. Nestor[1], and Yu-Chih Lin[1,*]

[1]Program in Neuroscience, Hussman Institute for Autism, Baltimore, MD-21201, USA
[2]Institute of Brain Science, School of Medicine, National Yang-Ming University, Taipei 112, Taiwan.
[3]Taiwan International Graduate Program (TIGP) in Molecular Medicine, National Yang-Ming University and Academia Sinica, Taipei, Taiwan.
[4] Brain Research Center (BRC), and Biophotonics and Molecular Imaging Research Center (BMIRC), National Yang-Ming University, Taipei 112, Taiwan.
[5] Program in Molecular Medicine, University of Maryland School of Medicine, Baltimore, MD-21201, USA

***Correspondence:** yclin@hussmanautism.org

Methods

Transfection and plasmids

For NIH/3T3 cells all transient transfection procedures were carried out acutely after plating cells using Lipofectamine P3000 (Life Technologies). iPSCs were transfected 24 hrs after plating using Lipofectamine Stem (Life Technologies). DNA plasmids used were pEGFP-C3 mCEP290 and pmCherry-C3-mCEP290 (gifts from Dr. Joseph Gleeson, Addgene plasmids #27379 and #27380), Ptc1-pEYFP N3 (gift from Dr. Matthew Scott, Addgene plasmid #58456), pGE-mSmo-YFP (gift from Dr. Philip Beachy, Addgene Plasmid #38094), pGL3b GliX8:EGFP (kindly provided by Dr. James K. Chen at Stanford University, CA, USA), Smo-EGFP, 5HT6-EGFP, SSTR3-EGFP, IA-EGFP (generously provided by Dr. Yu-Chun Lin, National Tsing Hua University, Taiwan), Arl13b-mCherry (gift from Dr. Xiaobing Yuan) and pmRFP-N1.

Cloning and Site-directed mutagenesis

Mouse Cep290 cDNA insert was amplified by PCR using the following PCR primer Cep290-Forward primer: TTGCTCGAGATGCCACCTAATATAAAGTGGAA (Xho1-Cep290); Cep290-Reverse primer: CGCCCCGGGCTAATAAATAGGGAAACTATGA (Sma1-Cep290). After purification the PCR fragment were ligated pCIG2 vector (from Dr. Olivier Ayrault, Curie Institute, France) digested with Xho1 and Sma1. D665G and R1747Q missense point mutations were introduced into pEGFP-Cep290 and pmCherry-Cep290 using the QuikChange II XL Site-directed mutagenesis kit (Agilent Technologies) and procedures were carried out according to the manufacturer’s instructions. Primers used were 5’-AAGGACATGCCGAAGGGTTCTGATGTGAAAGGAG-3’ (D665G, forward), 5’-CTCCTTTCACATCAGAACCCTTCGGCATGTCCTT-3’ (D665G, reverse), 5’- CAAAAGGCACTTAGTCAAG CCCTGTTGGAACTTC-3’ (R1747Q, forward), 5’-GAAGTTCCAACAGGGCTTGACTAAGTGCCTTTTG-3’ (R1747Q, reverse). Confirmation of single nucleotide exchange was performed by DNA sequencing at the Genomics Core Facility at the University of Maryland School of Medicine.

Immunoblotting

Mouse brain tissue and cell line cultures were lysed in RIPA buffer (Cell signaling technologies) containing PMSF and 8 mg/ml MS-SAFE^{TM} protease and phosphatase inhibitor cocktail (Sigma-Aldrich). Protein concentration was determined by BCA method (Thermo Fisher). Lysates (10 μg/lane - brain tissue; 50 μg/lane - cell sample) denaturated with NuPAGE LDS sample buffer were separated in NuPAGE Tris-Acetate 3-8% gradient gel (Thermo Fisher). Proteins were blotted onto PVDF membrane (BioRad), blocked with 5% non-fat milk in TBS-T. Membranes were routinely cut at around either the 117 or the 171 kDa marker (HiMark pre-stained protein standard, Life Technologies) and upper part was incubated with primary antibodies rabbit anti-mCherry (Thermo Fisher) or rabbit anti-CEP290 (Bethyl laboratories Inc.), while lower part was incubated with either rabbit anti-GAPDH (clone 14C10, Cell Signaling Technology) or mouse anti-β-tubulin III (clone 2G10, Sigma-Aldrich). Goat anti-rabbit IgG and goat anti-mouse IgG HRP-conjugated secondary antibodies (Life Technologies) were diluted in TBS-T. Proteins were detected using Clarity Western ECL substrate (BioRad) and chemiluminescence signal was documented using ChemiDoc^{TM} Touch Imaging system (BioRad). Optical density measurements were performed in Image Lab 5.2.1 (Bio Rad).

Figure legends

Figure S1 (related to Figure 1). Primary cilia morphology in serum-stimulated NIH/3T3 cells. Cells transfected with indicated plasmids were grown in bovine calf serum-supplemented culture medium and stained with antibody against acetylated tubulin to detect presence of primary cilium. **(A)** Fractions of cilia bearing cells in the transfected cell populations were determined from three experiments, $n \geq 120$ cells for each group. Data are represented as mean ± SEM. $*p < 0.05$, one-way ANOVA *post hoc* Holm-Sidak's multiple comparisons test. **(B)** Cumulative frequency distributions of cilium lengths. Analysis was performed by acquiring 6-10 μm Z-stacks of EGFP positive cells and axoneme lengths were thereafter measured in the ZEN desk 2012 software ($n \geq 72$ cells/group, three experiments, $****p < 0.0001$, Kolmogorov-Smirnov t-test).

Figure S2 (related to Figure 3). Smoothened localisation in NIH/3T3 after SAG treatment. (A) Quantification of the different Smo localisation pattern in cells cotransfected with Smo-YFP and indicated proteins after treatment with vehicle (ddH_2O) or SAG (400 nM) for 2 hrs prior to fixing and immunocytochemical procedures. n ≥ 160 cells per group and treatment, three independent experiments. Data are represented as ± SEM.

Figure S3 (related to Figure 4). Subcellular localisation of ciliary receptors and Cep290 constructs in cells used in FRAP analysis. Representative images of NIH/3T3 cells used in FRAP assay. Cells were cotransfected with vector or CEP290 constructs together with **(A)** Smo-EGFP, **(B)** 5HT6-EGFP, **(C)** SSTR3-EGFP, **(D)** IA-EGFP, and **(E)** Arl13b-mCherry. Cells were starved for 24 hrs prior to live cell imaging and photobleaching procedures. White arrows point to the localisation of Cep290 constructs at the base of cilia. Scale bar = 5 μm.

Figure S4 (related to Figures 1 and 6). Western blots used to generate cropped images. (A-C) NIH/3T3 cell lysates; see Figure 1B. **(D-E)** Mouse brain lysates for temporal analysis; see Figure 6A. **(F-G)** Mouse brain lysates for spatial analysis; see Figure 6B. Reference molecular weights in kDa were determined from the HiMark Pre-stained protein standard.

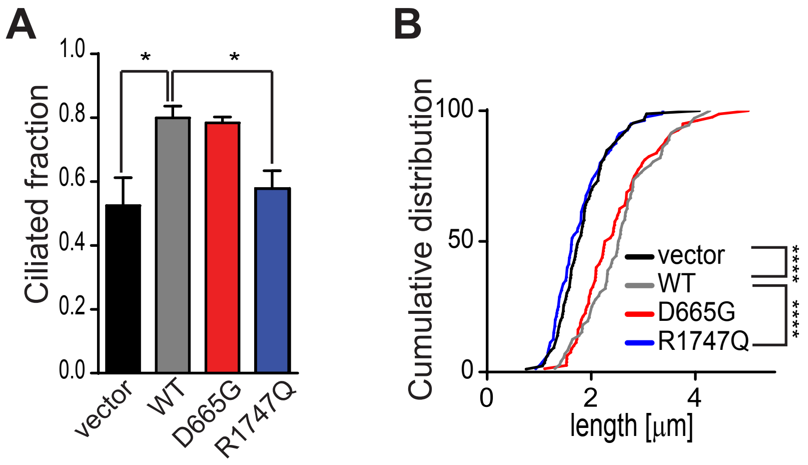
A
B
*
*
1.0
0.8
0.6
0.4
0.2
0.0
Ciliated fraction
vector
WT
D665G
R1747Q
Cumulative distribution
100
50
0
vector
WT
D665G
R1747Q
****
****
0
2
4
length [μm]

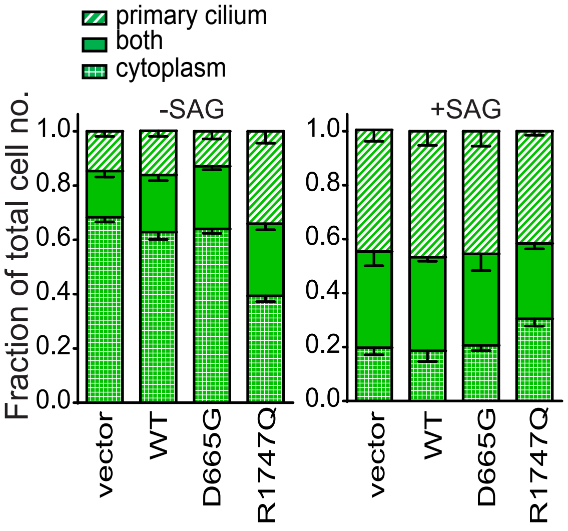
primary cilium
both
cytoplasm
-SAG
+SAG
Fraction of total cell no.
1.0
0.8
0.6
0.4
0.2
0.0
vector
WT
D665G
R1747Q
vector
WT
D665G
R1747Q

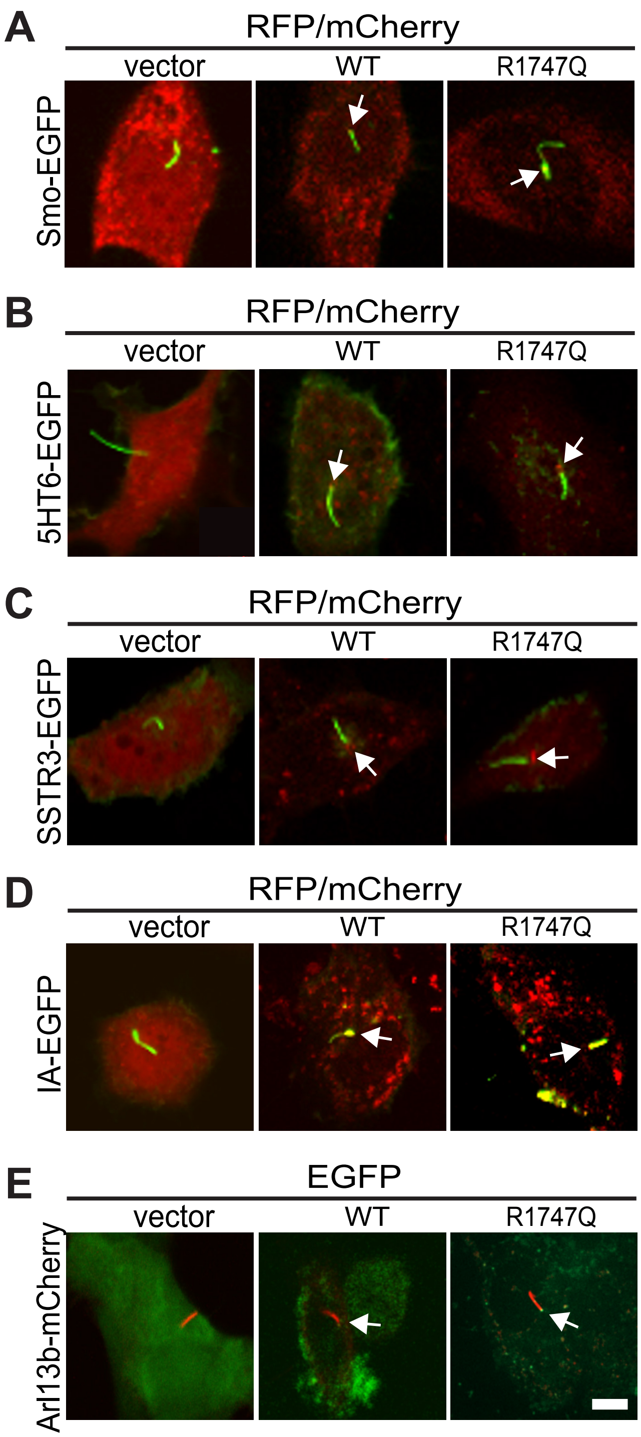

A
RFP/mCherry
vector
WT
R1747Q
Smo-EGFP
B
RFP/mCherry
vector
WT
R1747Q
5HT6-EGFP
C
RFP/mCherry
vector
WT
R1747Q
SSTR3-EGFP
D
RFP/mCherry
vector
WT
R1747Q
IA-EGFP
E
EGFP
vector
WT
R1747Q
Arl13b-mCherry

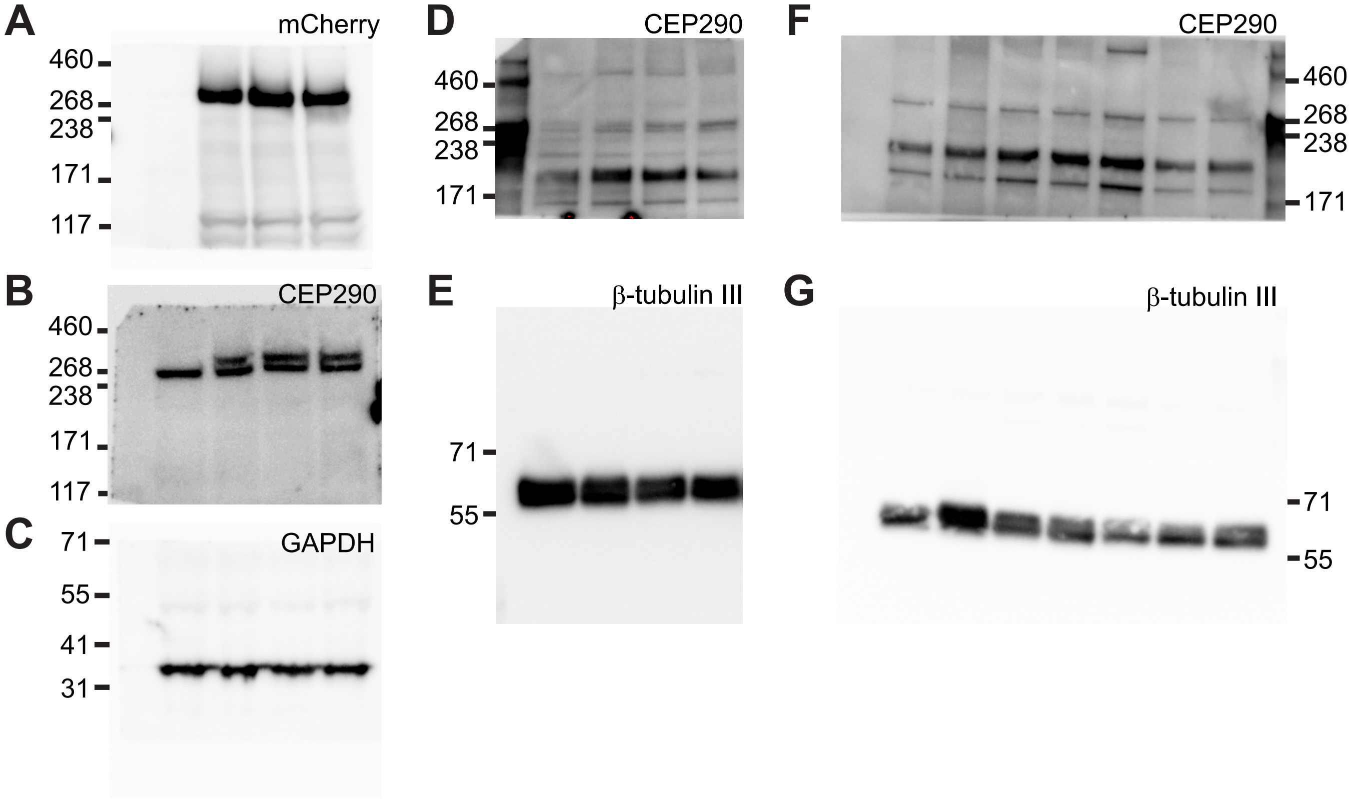
A
mCherry
460
268
238
171
117
B
CEP290
460
268
238
171
117
C
GAPDH
71
55
41
31
D
CEP290
460
268
238
171
E
β-tubulin III
71
55
F
CEP290
460
268
238
171
G
β-tubulin III
71
55
